# Sex differences and sex-specific regulation of motivated behavior by Melanin-concentrating hormone: a short review

**DOI:** 10.1186/s13293-024-00608-0

**Published:** 2024-04-03

**Authors:** Isabel R. K. Kuebler, Mauricio Suárez, Ken T. Wakabayashi

**Affiliations:** 1 Neurocircuitry of Motivated Behavior Laboratory, Department of Psychology, University of Nebraska-Lincoln, Lincoln, NE 68588-0308 USA; 2 Rural Drug Addiction Research Center, University of Nebraska-Lincoln, 660 N 12th St., Lincoln, NE 68588 USA

**Keywords:** Melanin-concentrating hormone, Motivated behavior, Sex, Lactation, Maternal behavior, Feeding behavior, Drugs of abuse

## Abstract

Recent preclinical research exploring how neuropeptide transmitter systems regulate motivated behavior reveal the increasing importance of sex as a critical biological variable. Neuropeptide systems and their central circuits both contribute to sex differences in a range of motivated behaviors and regulate sex-specific behaviors. In this short review, we explore the current research of how sex as a biological variable influences several distinct motivated behaviors that are modulated by the melanin-concentrating hormone (MCH) neuropeptide system. First, we review how MCH regulates feeding behavior within the context of energy homeostasis differently between male and female rodents. Then, we focus on MCH’s role in lactation as a sex-specific process within the context of energy homeostasis. Next, we discuss the sex-specific effects of MCH on maternal behavior. Finally, we summarize the role of MCH in drug-motivated behaviors. While these topics are traditionally investigated from different scientific perspectives, in this short review we discuss how these behaviors share commonalities within the larger context of motivated behaviors, and that sex differences discovered in one area of research may impact our understanding in another. Overall, our review highlights the need for further research into how sex differences in energy regulation associated with reproduction and parental care contribute to regulating motivated behaviors.

## Introduction

Melanin-concentrating hormone (MCH) is a 19 amino acid cyclic peptide originally isolated from the pituitary of salmon [40] and then discovered in mammals in 1989 [68, 95]. Several different lines of research have revealed that MCH regulates a broad range of neurobehavioral functions. For example, the role of MCH has been studied in a variety of areas such as energy homeostasis, sleep-wakefulness, and the regulation of eating, as well as the ingestion of alcohol [7, 22, 24, 25, 30, 70, 76]. Neuroanatomically, MCH-producing neurons are found in the lateral hypothalamic (LH) and the incertohypothalamic (IH, otherwise known as the zona incerta) areas of rats of both sexes [11, 12, 88], where they project throughout the brain, reflecting these diverse functional roles of the hypothalamus [16, 20, 22, 35, 78]. Like other circuits in the hypothalamus, subtle variations in MCH neuron expression and function may have outsized roles in sex-specific behaviors [83, 96]. Uniquely, neurons that transiently express MCH have recently been discovered in females. These neurons appear to be important for the expression of maternal behavior, which will be discussed in more detail below. Terminal projections of constitutively MCH-producing neurons are found in the nucleus accumbens (NAc) and the ventral tegmental area, key areas within the mesolimbic reinforcement circuit, a system that regulates the motivation to seek and consume natural and drug-specific rewards [20]. These neural projections exert their effect at two types of MCH receptors, melanin-concentrating hormone receptor 1 and 2 (MCHR1 & MCHR2). While MCHR1, a G-protein coupled receptor, is found ubiquitously across vertebrates including humans, MCHR2, while present in humans, is not found in either rats or mice [91].

### MCH and feeding behavior

The effects of MCH activity on feeding behavior have been extensively studied [22, 31, 77], with increasing evidence revealing sexual dimorphism in both physiology and behavior. Experiments manipulating MCH activity by directly increasing MCH neuron firing or changing the activity of MCH receptors show that the MCH system regulates feeding behavior. For example, Santollo and Eckel [79] found that broadly stimulating central MCH receptors using intracerebroventricular (ICV) injections of MCH increases meal size in gonadally intact male rats and non-intact ovariectomized female rats. Importantly, this increase in meal size in ovariectomized females is attenuated by 17-beta estradiol administration, a treatment that reintroduces circulating ovarian estradiol. This manipulation suggests that this estrogen lessens the orexigenic (i.e., appetite-increasing) effect of MCH. Indeed, the effect of MCH on feeding in intact free-cycling females is observed more robustly in diestrus 2 before the highest-estrogen phase of the ovarian cycle (i.e., proestrus), compared to after the high-estrogen phase during estrus [79]. This highlights the possible contribution of the pharmacokinetic and pharmacodynamic effects of estradiol. That is, the behavioral effects of MCH on feeding may be offset from when peak estradiol levels are observed. Time and cycle-dependent effects of sex steroids on feeding are well-established, as the effects of sex steroids have been observed to occur hours to days after their administration [27, 81, 85]. Thus, these two experiments enticingly suggest that the orexigenic effect of MCH is influenced by estradiol. Presently, the precise actions of estrogens on MCH-regulated feeding are only now beginning to be further delineated with more modern anatomically targeted approaches.

For example, Terrill and colleagues [93] have reported mesolimbic site-specific differences between males and females in MCH-induced feeding behaviors. Specifically, both pharmacological activation of MCH receptors and chemogenetic activation of MCH neuron terminals in the shell region of the NAc increase both chow and sucrose pellet feeding for male but not intact female rats. These increases were independent of changes in other behaviors often regulated by the NAc, such as overall locomotor activity, wall-hugging thigmotaxis (an index of anxiety-like behavior), or effort-based choices between high-effort sucrose pellets or freely available chow. Therefore, the authors of this study advance the idea that the general hyperphagia induced by accumbal MCH is not attributable to non-specific effects on NAc function. However, given that MCH has also been shown to influence cue-induced drug seeking behavior [18] it will be important to determine if these feeding-related MCH effects are the result of altered incentive salience and Pavlovian processes, another major functional role of the NAc.

More germane for this discussion, Terrill and colleagues [93] also reported that ovarian hormones influence the sexually dimorphic orexigenic effects of MCH, specifically in the NAc. When researchers administered MCH into the NAc of intact or ovariectomized female rats, they observed that MCH would increase feeding in the vehicle control condition in ovariectomized but not intact female rats. That is, ovariectomized females expressed similar behavior to male rats. This increased feeding effect was attenuated in ovariectomized female rats after a synthetic estradiol replacement treatment. Therefore, since the vehicle treated ovariectomized female rats had a similar response as males to MCH, and estradiol hormone replacement returned these ovariectomized females to their free-cycling behavior, the results suggest that circulating estradiol decreases the efficacy of MCH in the NAc to induce feeding in intact female rats.

This hormone-dependent activity may not simply be induced by the presence of more circulating estrogens, that is, the pharmacokinetic effects of hormones, but in fact may be also regulated by the number of estrogen receptor-1 (ER1) in the NAc shell—a pharmacodynamic effect. To further delineate possible physiological mechanisms for this NAc-specific MCH, estrogens, and sex interaction, Terrill and colleagues [93] assessed whether there was a preponderance of MCHR1 or ER1 in the NAc. They reported that both sexes expressed similar levels of MCHR1, consistent with another group’s findings in the same brain region [21], but that females expressed significantly more ER1 in the NAc. In addition, female rats had more neurons that coexpressed both MCHR1 and ER1 [93]. Estrogens may affect feeding indirectly by changing the expression of MCH. Santollo and Eckel [80] found that increased estradiol, in both natural and experimenter-manipulated conditions, results in decreased MCH expression in the LH. The authors hypothesized that estradiol may act on ER1 in MCH cells, as ER1 is the primary estrogen receptor that regulates feeding [81]. However, MCH neurons in the LH do not coexpress ER1 in either male [66] or female rats [80]. Therefore, the authors suggest that estradiol acts on a local network of neurons in the LH to decrease the expression of MCH neurons [80]. It is currently unknown whether MCH neurons express the other estrogen receptor subtype ER2, but this estrogen receptor likely does not regulate feeding [81]. More broadly, while the patterns of either MCH peptide expression [28], or the gene that encodes its preproprotein, *pmch* [9, 86] appear similar between the male and female rodent LH and IH, it is currently unknown whether the total number of MCH expressing neurons differs between the sexes. Together, the diverse anatomical targets of MCH neurons, their receptor distribution, and the wide impact of pharmacodynamic effects of estrogens on MCH-mediated feeding, emphasize the importance of localizing the effects of MCH to specific brain regions to better resolve the contributions of sex and hormones towards behavior.

### MCH activity in response to glucose and insulin

Broadly speaking, feeding behavior is regulated using sensory and physiological cues to balance total energetic resources available to the organism. Therefore, in addition to considering MCH from a mesolimbic motivational neurocircuit perspective, it is also important to consider that MCH neurons regulate feeding behavior via their role in physiological energy regulation. Many neurons, like those that release MCH, can regulate behavior by directly sensing brain glucose levels; glucose serves as the primary form of fuel for the brain, and central glucose detection is a critical function for a variety of nutrient-detecting neurons (for an in depth review, see [62]). Routh and colleagues [77] hypothesized that one purpose of glucose-sensing neurons is to protect the brain’s main energy supply during periods of low blood glucose, such as starvation. This protection may be provided by directing energy away from non-critical bodily functions, such as reproduction, especially in females. For example, in humans, menses is delayed or paused as a result of low energy stores, known as anorexic amenorrhea [36, 72]. However, this ability to allocate energy resources may extend beyond moments of extreme physiological distress. Mechanistically, MCH neurons are activated by the closure of ATP-sensitive potassium channels in the presence of glucose [44]. Burdakov and colleagues [13] found that in male mice, increasing the concentration of extracellular glucose from a hypo- to a hyperglycemic state dose-dependently depolarized MCH neurons. They also found that when applying a hyperpolarizing current, MCH neurons hyperpolarize less in the presence of glucose, showing increased membrane resistance to changes in potential compared to lower glucose levels. Overall, these findings establish that the electrical excitability of MCH neurons in vitro and in vivo increases at higher glucose concentrations in male mice. Presumably this finding extends to female mice, but this has yet to be empirically determined.

However, another group reports that LH MCH neurons are active during periods of low glucose. Mogi and colleagues found that for both sexes, fasting resulted in high levels of phosphorylated cyclic adenosine 3′,5′-monophosphate response element-binding protein (pCREB), a biochemical marker correlated with increased neuronal firing [63]. In this study, these high levels of MCH activity were reduced by an injection of glucose after 15 min, similar to fed animals. A possible reason for the disparity between electrophysiological and biochemical indices of activation is that these measures occur at different timescales (e.g. milliseconds versus minutes) and capture different processes. Evaluating changes further along the biochemical pathway may provide more insight into the final output state of these MCH neurons and provide a more direct link between the temporally rapid electrophysiological and slower biochemical data.

MCH neurons also respond to insulin levels in the brain to regulate feeding and energy homeostasis. Insulin is a critical hormone that is released from beta cells of the pancreas after feeding to promote cellular intake of blood glucose. Ludwig and colleagues [58] found that male mice with overexpressed MCH and eating a high fat diet ate more food and had higher body weight than wild-type controls eating the same diet. Mice with overexpressed MCH also had a blunted insulin response and thus overall higher blood glucose levels after a glucose injection [58]. Hausen and colleagues [33] later investigated the mechanism of insulin’s regulation of MCH. They found that insulin increases the excitability of a subset of MCH neurons, depending on the activity of the insulin receptor. High fat diet-fed male mice with chronically inactivated insulin receptors in MCH neurons showed improved insulin sensitivity and higher locomotion than controls. This suggests that insulin impacts MCH neuronal signaling and may contribute to long-term changes in glucose metabolism and energy expenditure, particularly under conditions modeling obesity.

### Glucose-sensing properties of MCH neurons vary across sex

MCH neuronal activity in response to glucose is also influenced by sex and gonadal hormones. Another goal of the Mogi study was to systematically determine the interaction of sex, circulating hormones, and feeding on MCH activity. A diagram summarizing results from this study is shown in Table 1.

**Table 1:** A synopsis of MCH neuronal biochemical activation (reported as phosphorylated cyclic adenosine 3′,5′-monophosphate response element-binding protein (pCREB) expression levels) after a glucose challenge in different experimental groups; results from [63]

Rat group	Experimental manipulation			
	Fed (saline)	Fasted (saline)	Fasted (glucose + 5 min)	Fasted (glucose + 15 min)
pCREB expression in lateral hypothalamic MCH neurons				
Intact male	Low	High	High	Low
Orchiectomized male	Low	High	Low	
Orchiectomized male + testosterone	Low	High	High	
Orchiectomized male + estrogen	Low	High	Low	
Intact female proestrus + estrogen	Low	High	Low	Low
Ovariectomized female	Low	High	High	
Ovariectomized female + testosterone	Low	High	Low	
Ovariectomized female + estrogen	Low	High	Low	

In fasted intact female rats, MCH neuronal activity returns to low levels after a glucose challenge (i.e., similar to fed intact female rats) faster than for the same manipulation in intact males. The authors suggest that higher amounts of testosterone cause MCH neurons to be less sensitive to rising blood glucose. This suggests that both gonadal hormones regulate MCH neurons’ glucose-sensing properties by adjusting their sensitivity to glucose. However, removal of the gonads in adult rats also shows differences in response across sex in the absence of circulating gonadal hormones. Ovariectomized female rats and orchiectomized male rats show opposite responses in MCH activity shortly after a glucose challenge. Activity in castrated males decreases more readily than intact males, an effect seen in intact females. Ovariectomized females sustain fasting activation levels, like intact males. This result supports the idea that developmental differences across sex are also critical for glucose-sensing functions [47], and that MCH neurons may also contribute to this process. Estrogen replacement in both sexes of gonadectomized rats allows MCH activity levels to quickly decrease to fed levels, similar to intact female rats. However, testosterone replacement decreases the sensitivity of MCH neurons only in castrated male rats. In female rats, testosterone replacement resembles estrogen replacement, increasing the sensitivity of MCH neurons. The authors speculate that testosterone replacement may affect MCH activity only in ovariectomized females through testosterone’s intracellular conversion to estrogens via aromatase, where female rats are more sensitive to the effect of estrogens while male rats are more sensitive to the effect of testosterone [63]. Mechanistically, while aromatase expression is generally restricted to neurons that strongly regulate reproductive behavior (e.g., mPOA, for review see [50, 75]) it is found throughout the LH [67]. Currently it is unknown whether aromatase colocalizes with MCH neurons, although it should be noted that MCH neurons also do not express ER1 receptors themselves. It remains possible that aromatase may influence the MCH system through local network interactions with other ER1 expressing LH neurons [67]. In all, the results of Mogi and colleagues’ landmark paper suggest that MCH neuron sensitivity to glucose is enhanced by ovarian hormones and attenuated by testicular hormones, and that fundamental physiological and developmental differences across sex also play a role in MCH glucose-dependent activity. Further, MCH may be a key factor in hormonal regulation of energy balance across sex and reproductive stage [36, 65]. This study highlights the importance of considering both these sex factors in the design of contemporary studies of MCH function in broader investigations of motivated behavior.

### Contributions of MCH to maternal physiology and behavior

A necessary requirement for the survival of a species is appropriate maternal behavior and the associated physiological adaptations needed to care for the young. However, maternal behavior is not a simple motor response but a constellation of behavioral phenomena such as the approach, adequate care, and eventual weaning of young. The successful rearing of mammalian offspring is dependent on the neural and hormonal conditions underlying pregnancy, parturition, and lactation, the generation and release of milk [46]. Particularly, pregnancy and lactation alters energy expenditure and requirements to provide for fetal tissue and milk production, through several hormonal pathways [6, 37, 65]. MCH regulates behaviors and physiological adaptations typically seen in dams (rat mothers) interacting with their young (pups). While male rodents can also exhibit these behaviors, most studies have focused on MCH’s impact on female maternal behavior, advancing the view that MCH plays a pivotal role in the neuroadaptations related to the unique energetic demands on females associated with supporting maternal behavior. For this discussion, we will first focus on lactation in the larger context of maternal behavior. This is a sex-specific process in females that is energy intensive, and normally only occurs just after dams have given birth.

### MCH and regulation of lactation by sex-specific mPOA MCH neurons

The link between MCH and lactation was first reported in 1992, when Knollema and colleagues discovered a unique population of neurons that transiently express MCH in female rats [42]. This unique population of neurons is located in hypothalamic subregions including the medial preoptic area (mPOA), periventricular (PeA), and paraventricular (PVN) nuclei only during lactation. Corresponding areas in males do not express MCH. These brain areas are known to influence a variety of functions in both sexes, including parental behavior, thyroid stimulation, and digestion [22]. Transient MCH expression seen in females varies with reproductive experience, it is highest during the first lactation, and lessens with subsequent litters, supporting the idea that there are experience-dependent neuroadaptations in female-specific MCH neurotransmitter systems [92]. Moreover, Knollema and colleagues report that mPOA and PeA expression of MCH is temporally correlated with the progress of the lactation period, as it is not seen in female rats sacrificed 4–8 days after pup weaning, during late pregnancy, or during estrous (nonpregnancy). Later studies showed that this MCH expression in the mPOA, the PeA, and anterior PVN has been observed up to 19 days after birth [19, 29]. Thus, transient MCH expression has a tight temporal correlation specifically with the cessation of lactation, since young rats are weaned at approximately 21 days.

The effects of these transient neurons on downstream projection areas may further uncover a mechanism for their role in lactation and maternal behaviors. Transient expression of MCH has been discovered in fibers projecting from the mediobasal subdivision of the mPOA to the posterior pituitary up to day 19 of lactation [19]. The posterior pituitary contains the terminals of oxytocin-producing neurons originating from the hypothalamus (see [41]). This oxytocin is responsible for the milk ejection reflex which allows suckling pups to access milk. These transient MCH-positive fibers show axonal varicosities near oxytocin terminals, which is neurostructural evidence of a site of neurotransmitter release. Therefore, transient mediobasal mPOA MCH projections may act an upstream regulatory input to oxytocin as a modulator of milk release [19]. Finally, as these circuit-level mPOA MCH cell bodies are no longer observable 4–8 days after pup weaning [92], it can be speculated that their projections to the posterior pituitary also wane after pup weaning. This reduction further implicates an adaptational role of MCH in reallocating energy resources and effort away from now unneeded maternal behavior and milk production, though this has yet to be verified experimentally.

If transient MCH expression is needed for the sex-specific energy demands of maternal care, such as lactation, one would predict that litter size would also correlate with the magnitude of this female-specific MCH expression. To test this, Ferreira and colleagues quantified MCH immunoreactive neurons of the mPOA 19 days postpartum in dams that had large or small litters and found that MCH neuron count positively correlated with the number of pups born. To test if the increase in MCH neurons was due solely to the birthing of more pups, Ferreira also artificially reduced litter size by removing pups on postnatal day 1. This resulted in MCH neuron counts that were similar to the naturally small pup group and significantly less than then large litter group [29]. Thus, this experiment supports the hypothesis that female-specific transient MCH neurons provide the neural substrate for the energetic demands specific to maternal behavior until pups begin to wean. These results demonstrate that sensory stimuli is correlated with the expression of transient MCH neurons, suggesting that MCH expression may be a necessary component driving maternal behavior.

Some groups have suggested that suckling from pups provides an exteroceptive stimulus that ultimately influences transient MCH expression in dams. However, the results of recent investigations directly testing this mechanistic hypothesis have been ambiguous. Estrogens are critical for the onset of maternal behavior, and neurons of the mPOA express ER1; these same cells are active during maternal behavior [55]. Additionally, neurons of the mPOA also contain oxytocin receptors, with oxytocin receptor binding reported to be more in rats that become maternal faster than those with delayed maternal onset [14], suggesting that these neurons are sensitive to pup-mediated stimulation. Using Fos protein immunoreactivity as a marker for neuronal activation, Alvisi and colleagues directly assessed whether suckling stimuli from pups induces Fos in neuroanatomical areas with robust sex-specific and sex-generalized MCH expression, such as the mPOA, IH and LH, when compared to a group where suckling was prevented. While overall Fos immunoreactivity is higher in all brain areas in dams that experienced pup suckling than those that had no suckling, Fos does not colocalize with MCH neurons [4]. Despite this, they found that MCH-immunoreactivity itself (i.e., the number of MCH neurons) in the IH and mPOA is impacted by the day of lactation, independent of suckling, peaking around the 15th–16th day (mPOA) and 17th and 21st day (IH). These numbers are consistent with the findings reported in previous studies that transient MCH expression is maximal around the 19th postnatal day. Yet, in the LH there is an interaction between postnatal day and suckling, so that rats without suckling stimuli have sharply decreased MCH expression compared to those with suckling on postnatal day 19. This means that suckling influences MCH expression in a sex-generalized area of the brain (i.e., LH) more than a sex-specific area (i.e., mPOA) depending on the postnatal day. In total, suckling from pups regulates MCH expression without direct Fos activation of MCH neurons. The authors suggest that pup-mediated Fos activation in the vicinity of MCH neurons may reflect activation of a local network of neurons within the LH. Therefore, exteroceptive stimuli may indirectly determine the number of MCH neurons in sex-generalized rather than sex-specific areas.

Litter size increases milk demand, and this demand increases the secretion of prolactin, a hormone critical in the regulation of maternal care [23]. Rat mPOA MCH neurons express prolactin receptor mRNA [43], and prolactin pretreatment in both mice and rats results in the upregulation of the phosphorylated signal transducer and activator of transcription 5 (pSTAT5) transcription factor, a measure of prolactin receptor activation [43]. The consequences of prolactin on the MCH system appear complex, both the presence and absence of prolactin have been reported to attenuate transient MCH expression. During lactation, systemic administration of prolactin, which activates the prolactin receptor, decreases MCH expression in the mPOA [92]. However, genetic knockout of the STAT5 gene, which inhibits prolactin receptor activation and functionally mimics blocking the effects of prolactin, also decreases MCH expression [92]. This reduction of transient MCH expression in the mPOA is also seen with the pharmacological reduction of circulating prolactin during lactation [43]. While these data implicate prolactin as a major factor regulating transient mPOA MCH neurons, further work will need to be undertaken to more precisely delineate the mechanistic effects of prolactin on transient mPOA MCH expression.

The influence of other hormones necessary for maternal care on transient mPOA MCH neurons has also begun to be explored, primarily through receptor expression. Teixeira and colleagues [92] discovered that the majority of transient MCH neurons express the ER1 receptor as well as prolactin-induced pSTAT5. They suggest that dynamic fluctuations in estrogens levels during pregnancy or postpartum play a role in the transitory expression of MCH in mPOA, preparing these neurons for supporting and regulating later maternal care. The contributions of estrogens with and without the influence of prolactin will need to be determined to understand all the factors necessary for the expression of transient MCH mPOA neurons.

It is clear that the transient expression of MCH in the mPOA relies on the contribution of multiple hormones, ongoing exteroceptive pup stimuli during lactation, and maternal experience. In these studies, the presence of prolactin and ER1 receptors on MCH neurons, sensitivity to STAT5 activation or deletion, and possible upstream modulation of oxytocin implicates MCH neurons as a critical neural integrator of the energetic demands of maternal behavior. Further, repeated experience raising pups demonstrates long-lasting, permanent changes to transient mPOA MCH neuron expression. In other words, these combined effects of hormones, physical stimuli, and reproductive experience provide a constellation of physiological and behavioral components that regulate the expression of MCH in the mPOA.

### MCH and modulation of active maternal behaviors by sex-specific mPOA MCH neurons

MCH in the mPOA also influences other maternal behaviors besides lactation. Functionally, MCH administration directly into the mPOA of female rats during early postpartum, dose-dependently alters litter grouping behavior. Specifically, while a low dose increases the latency of dams to group the entire litter, a higher dose of MCH significantly inhibits this behavior as well as other aspects of maternal care [10]. These other high dose MCH effects include reductions in the number of individual pup retrievals, mouthing, and nest building. This manipulation demonstrates that direct MCH receptor activation via MCH itself in the mPOA is sufficient to mimic the effects of increased mPOA MCH expression seen during late lactation and as maternal behavior begins to subside [42, 74]. Surprisingly, systemically interfering with the action of MCH in the entire mouse brain has an effect similar to activating MCH receptors via direct microinjection of MCH in the mPOA. For example, systemic administration of the MCHR1 antagonist GW803430 in mice during early postpartum decreases maternal behavior and lactation [2]. This includes lower frequencies of nest building, pup retrieval, and maternal aggression toward other mice. The reduction of maternal behavior after both stimulating and inhibiting MCH receptors could potentially be explained in two ways. First, these similar behavioral effects from contrasting manipulations may be the result of brain circuit-level effects. Recall that MCH is also constitutively expressed in LH and IH areas as well as transient expression in the mPOA, and that MCH neurons project widely throughout the brain and functionally impact a wide range of behaviors and processes beyond that of maternal behavior. Thus, the behavioral effects of brain-wide MCH manipulations may be the result of competing, non-specific effects in circuits or brain areas not directly involved in maternal behavior. Moreover, a brain-wide genetic knockout may give rise to compensatory neuroadaptations over the development of the mouse. Like feeding behavior, short-term localized changes in MCH receptor activation may show results different from a longer blockade of MCH receptor function. Second, as maternal behavior is dynamic and occurs in distinct temporally defined phases of initiation, maintenance, and decline [53], the timing of brain MCH manipulations may affect some individual behavioral components stronger than others. During the initiation phase, maternal care expressed by the dam is highly motivated and responsive to the physical characteristics of the pups. This occurs during a period when mPOA MCH expression is relatively low, and a further reduction of MCH activity here may reduce the impact of early pup stimuli, preventing subsequent care. In support of this, Alachkar and colleagues [2] reported greater mortality of pups on postpartum day 1–2 birthed by MCH knockout mice with no MCH expression. Although, by three days postpartum, infant mortality rate was no different between MCH knockout and wild-type dams, suggesting this is a phenomenon unique to the earliest phases of maternal initiation. On the other hand, Benedetto increased MCH activity by directly infusing MCH into the mPOA on postpartum day 5–6, during the maintenance phase of maternal behavior, and reported a decrease in maternal care. During this time, mPOA MCH is temporally correlated with lactation, and exogenous MCH administration may mirror the decline of maternal care seen during weaning.

Infusing MCH into the brain may result in very high concentrations of MCH, leading to concerns about neurotoxicity. However, to date, there has been no quantification of physiological MCH levels in the study of maternal behavior beyond comparative in situ hybridization studies in female rats across several estrus and reproductive states [42]. With feeding, studies report a wide range of 1.3–379 fmol/mg of MCH per brain tissue depending on the brain area and MCH quantification method [49, 97]. The injections into the mPOA [10] are site specific and several orders of magnitude greater in concentration than physiological estimates, but do not appear to have post-treatment behavioral effects indicating neurotoxicity.

Taken together, altering mPOA MCH receptor binding or genetic knockout of MCH disrupts maternal behavior. This suggests that transiently expressed MCH function is integral to the initiation and decline of lactation and maternal behavior. The complex effects of MCH manipulations may be due to dynamic roles of these transient neurons over the course of the postpartum period. In future research, using chemogenetic or optogenetic technology to flexibly alter neuronal firing at different timepoints postpartum would be an advantageous way to determine the function of these MCH neurons and their downstream impact as a function of time.

### Maternal and analogous parental behaviors, MCH, and olfaction

Olfaction is a critical sensory modality in rodents because it plays a key role underpinning complex motivated behaviors related to foraging, social interaction, anti-predator behaviors, and parental behaviors [52, 82]. Evidence supporting the regulation of olfactory-mediated behavior by MCH has only been recently reported. For example, MCH knockout mice are less likely to find buried food or spend time in a zone where food has been buried compared to wild-type controls [1].

As maternal behavior is also reliant on olfaction, Alhassen and colleagues [3] evaluated if MCH disruption of maternal behavior occurred through that sensory modality. They reported an impaired development of maternal behavior in pup-naïve mice that were either constitutively MCH-deficient from birth (i.e., brain-wide knockout), or mice with a conditional MCH knockout localized to the LH and IH. Specifically, both MCH-impaired groups were observed to have longer onset pup retrieval and longer retrieval durations. Additionally, when given either an empty cup or a cup full of pups, both MCH-impaired groups showed no preference in time spent with the cup of pups, with the conditional MCH knockout even showing pup avoidance compared to wild-type controls. A key behavioral difference between full and conditional MCH knockout rats reported by Alhassen et al. was the inability of the full MCH knockout rats to appropriately respond to novel non-pup related smells, such as banana and lemon. The generalized effect of the full MCH knockout on non-pheromonal odorants suggests MCH is critical for the development of the olfactory system from birth. Locally disrupting MCH in the LH later in life may affect processing of species-specific pheromonal odorants alone, suggesting that the hypothalamic response to odorants is integral to parental behaviors and social interaction.

### Modulation of maternal and parental behaviors by sex-generalized LH MCH neurons

Recently, Kato and colleagues have shown through an elegant series of genetic targeting studies that LH MCH neurons projecting to oxytocin neurons in the PVN can directly regulate specific maternal and analogous parental behavior in unmated female and male mice, respectively. First, they found that genetically targeted conditional lesions of MCH neurons in both sexes resulted in mice showing less interest in pups. Next, they found that stimulation of MCH neurons in the LH by chemogenetics or optogenetics increased nursing behavior, and that parental nursing behavior corresponded with increased activity in these neurons [39]. Table 2 provides a summary of maternal behavior changes after MCH manipulation. Overall, disrupting MCH expression inhibits pup-stimulated parental behaviors, and enhancing MCH function supports parental behaviors toward pups. MCH’s involvement in maternal and parental behaviors prompted by olfaction alone suggests that olfaction is a major mechanism by which MCH regulates maternal behaviors.

**Table 2:** A comparison of maternal and parental behaviors after MCH and MCHR1 manipulations in female and male rodents

Behavior	Species	Sex	Area	Manipulation	Effect	References
Pup retrieval	Mice	Female, unmated	Systemic	MCHR1 knockout	Decreased	[3]
Pup retrieval	Mice	Female, unmated	Systemic	MCH knockout	Decreased	[3]
Pup retrieval	Rats	Female, postpartum	mPOA	MCH microinjection	Decreased	[10]
Pup interaction	Mice	Female, unmated	Systemic	MCHR1 knockout	Decreased	[3]
Pup interaction	Mice	Female, unmated	Systemic	MCH knockout	Decreased	[3]
Pup mortality	Mice	Female, postpartum	Systemic	MCH knockout	Increased	[39]
Pup mortality	Mice	Female, postpartum	Systemic	MCH knockout	Increased	[1]
Pup aggression	Mice	Male, unmated	Systemic	MCH knockout	Increased	[39]
Intruder aggression	Mice	Male, unmated	Systemic	MCH knockout	Increased	[39]
Crouching	Mice	Female, unmated	Systemic	MCH ablation	Decreased	[39]
Crouching	Mice	Male, unmated	Systemic	MCH ablation	Decreased	[39]
Crouching	Mice	Female, unmated	LHA	MCH chemogenetic activation	Increased	[39]
Crouching	Mice	Male, unmated	LHA	MCH chemogenetic activation	Increased	[39]
Crouching	Mice	Female, unmated	LHA	MCH optogenetic activation	Increased	[39]
Crouching	Mice	Male, unmated	LHA	MCH optogenetic activation	Increased	[39]
Crouching	Mice	Female, unmated	Systemic	MCH knockout	Decreased	[39]
Hovering over, nursing posture, and time with pups	Rats	Female, postpartum	mPOA	MCH microinjection	No change	[10]
Nest building	Rats	Female, postpartum	mPOA	MCH microinjection	Decreased	[10]

### The biochemistry of sex-specific transient MCH neurons

Describing the biochemical identities of a neural circuit helps reveal whether component neurons excite or inhibit downstream neural systems and may help in uncovering their functional role in regulating behavior. In the context of transiently expressing MCH neurons in the mPOA, early work has found that these neurons express mRNA for glutamic acid decarboxylase-67 (GAD-67), an enzyme that converts glutamate to γ-aminobutyric acid (GABA), suggesting that these neurons co-release the inhibitory neurotransmitter GABA [74]. However, more recent studies using a transgenic mouse approach labeling vesicular GABA transporter (VGAT), another key enzyme necessary for GABA synaptic release and neurotransmission, failed to verify that these mPOA MCH neurons co-express the synaptic transporter during late lactation [92]. The authors propose that GABA synthesized by GAD-67 is released through non-canonical pathways. For example, in other GABA systems, there is evidence of VGAT-independent GABA release in dopaminergic neurons, via VGLUT and possibly VMAT2-dependent mechanisms [94]. While it is reasonable that a similar alternative mechanism for GABA may exist in transient MCH neurons of the mPOA, more direct evidence is needed to support this hypothesis. A possible alternative hypothesis is that GAD-67 in these MCH neurons synthesizes GABA for uses other than neurotransmission [71, 89]. Additionally, the same study did not identify these transient MCH neurons as either glutamatergic or dopaminergic, nor do they express oxytocin, implicated in maternal behavior, or kisspeptins, neuropeptides important in reproduction.

In summary, transiently expressed MCH neurons in the mPOA may influence downstream functions by co-releasing GABA and inhibiting neural systems, and they respond to hormones important for maternal behavior and lactation. While these studies support the hypothesis that these neurons regulate maternal behavior, the exact function of these neurons and impacts on behavior will need to be delineated in the future.

### Sex differences, MCH, and drugs of abuse

In addition to motivated behaviors such as feeding and maternal behavior, there is mounting evidence that the MCH system also regulates the behavioral effects of drugs of abuse and symptoms related to substance use disorders. The lateral hypothalamus was first identified as a structure of interest in the context of substance use disorders when Olds [69] reported that rats would continually electrically self-stimulate the LH. The LH contains dopaminergic axons within the median forebrain bundle whose cell bodies originate in the ventral tegmental area (VTA), and terminate in the NAc (i.e., the mesolimbic dopamine pathway) [32, 45]. Recently, several researchers have reported neuroanatomical evidence of MCH regulation of the mesolimbic dopamine system. Saito and colleagues [78] found MCHR1 in the NAc, and Diniz and colleagues [20] described MCH terminal projections in both the NAc and VTA. These connections to the mesolimbic system suggest a role for MCH in reward processing and by extension, substance use disorders. Indeed, genetically deleting MCHR1 in GABAergic neurons of the NAc results in a hyperdopaminergic state [15]. The investigation into the exact mechanisms by which MCH affects symptoms of substance use disorder is still being actively investigated. Within substance use disorder etiology, MCH has been most extensively explored in the context of alcohol use disorder (AUD), where we will focus most of our discussion here.

#### MCH, alcohol seeking and intake

Broadly, MCH potentiates seeking and drinking of alcohol, which is also a caloric food. The effects of MCH on alcohol intake were first characterized in 2005 where a series of studies showed ICV administration of MCH which activates MCHR1 throughout the brain increases alcohol drinking [24]. Additionally, systemic administration of the MCHR1 antagonist GW803430 attenuates alcohol self-administration, suggesting that blocking MCHR1 activity inhibits the operant reinforcing properties of alcohol during alcohol self-administration [18, 38]. These studies demonstrate that pharmacologically manipulating the activity of MCH receptors directly impacts alcohol consumption and seeking. In contrast, genetic knockout of MCHR1 from birth results in overall increased alcohol consumption compared to wild-type controls [26]. This apparent contradiction in functional effects of MCH receptor manipulations depending on the method broadly parallels results seen in maternal behavior studies that are discussed earlier in this review. Therefore, we can extend the same speculation to MCH and drugs of abuse, namely that short-term MCH receptor activation may potentiate alcohol consumption, but a permanent absence of MCHR1 activity may lead to compensatory mechanisms elsewhere in the wider circuit network resulting in the same functional outcome.

In addition to modulating intake of alcohol, the genetic expression of MCH may also be impacted by experience with alcohol. Increased hypothalamic MCH expression is correlated with increased alcohol consumption [70]. The length of experience with alcohol has contrasting effects on MCH neuronal expression. Acute alcohol intake results in heightened MCH expression in the LH, while chronic alcohol experience dose-dependently reduces MCH expression [64]. The authors suggest that alcohol initially stimulates MCH expression, but chronic drinking engages a negative feedback loop that further reduces alcohol intake. The increased expression of MCH neurons supporting alcohol drinking has interesting parallels to the dynamic expression of MCH in the mPOA in lactating dams. The hypothesis that the MCH system regulates caloric alcohol drinking and maternal behavior through a common mechanism of homeostatic energy regulation is an intriguing possibility that would need to be empirically tested.

Despite this work, sex differences in how MCH regulates alcohol drinking has not been systematically studied, though there is a large body of evidence of sex differences in alcohol consumption in both animals and humans. For example, alcohol intake in rats has large differences across sex, females in general show increased vulnerability to the effects of alcohol. This difference was first shown in 1984 with rats given free choice between water and 10% alcohol bottles, with females preferring alcohol more than males and consuming more alcohol per body weight [54]. More recently, Randall and colleagues [73] found that while female rats drank more alcohol than male rats, male rats lever-pressed for alcohol more than female rats. The authors propose that alcohol is more positively reinforcing to male rats when part of an operant behavior, as opposed to free-drinking conditions with less response requirements. Therefore, sex differences here may reflect motivational differences for alcohol seeking rather than alcohol drinking. Considering the role of MCH in alcohol drinking, as an operant reinforcer for alcohol self-administration, and sex differences in other motivated behaviors, future research will need to carefully consider sex differences when studying MCH in preclinical models of alcohol use and use disorder.

#### MCH and other drugs of abuse

Beyond alcohol, the interaction between MCH and cocaine and methamphetamine has only been studied in male rats. First, MCHR1 antagonism inhibits operant responding for cocaine as well as reinstatement after extinction, suggesting that MCH regulates operant reinforcement for cocaine, as well as cue- and cocaine-induced relapse-like behavior [17]. Second, MCHR1 antagonism within the NAc potentiates the development of locomotor sensitization to methamphetamine, a behavioral symptom of chronic drug experience that has been linked to increased motivation for the drug as well as increased craving for drug and vulnerability towards relapse [5, 57, 87, 90].

With regards to nicotine, we have recently discovered a link between MCH, locomotor sensitization, and sex differences. We found that systemic MCH receptor antagonism during the expression of nicotine locomotor sensitization after chronic pretreatment and during drug withdrawal has different effects in male and female rats [48]. Specifically, a lower dose of the MCH antagonist GW803430 was effective at attenuating the expression of nicotine locomotor sensitization more in females than males. Our findings provide some of the first empirical pre-clinical evidence in adult rats that MCH regulates the behavioral symptoms of nicotine experience, indeed of any abused drug experience, differently when sex is considered as a factor. Further, this study suggests that targeting MCH receptor activation may be a promising therapeutic target for tobacco use disorder in humans.

Currently there are no reports investigating the role of MCH in opioid use disorder. However, both opioid receptors and MCHR1 are found on neurons in the NAc [60, 78], suggesting that MCH may regulate the rewarding properties of opioids [56]. It will be important to include sex differences in future research on MCH and opioids, as there are known differences in opioid behavioral symptoms across sex (for a review, see [8]). For example, female rats self-administering heroin show faster acquisition of drug taking than male rats [59], but male rats take more oxycodone during early acquisition than female rats [61].

#### The interaction of MCH, maternal behavior, and substance use disorders

Considering the importance of MCH in regulating sex-specific maternal behavior, another key interaction that will need to be investigated systematically is the intersection of MCH, maternal behavior and substance use disorders. This is because female rodents respond to drugs of abuse differently during pregnancy and lactation than when not raising pups. For example, decreases in both nicotine self-administration and nicotine locomotor activation have been reported in either pregnant or lactating rat dams when compared to controls [51]. As well, pregnant dams show decreased motivation to work for cocaine but not sucrose under a progressive ratio schedule, and only partially return to normal motivation during lactation [34]. Further, the incentive salience of cocaine is reduced during early but not late post-partum dams, as early post-partum dams prefer chambers associated with cocaine exposure much less than late post-partum dams [84]. These studies suggest that pregnancy and lactation are protective against the motivational and behavioral symptoms of drugs of abuse. Considering MCH’s dual roles in regulating maternal behavior and drug-seeking and taking, it is possible that the transient, sex-specific expression of MCH is the mechanism by which this change in motivation for drug seeking and taking behavior occurs.

Together, MCH may play a large part in sex differences of substance use disorder related behaviors. First, there are wide differences in patterns of drug use across sex and reproductive status in both animals and humans. Additionally, MCH regulates feeding and other motivated behaviors differently across sex. These interactions suggest that MCH regulates the effects of drugs of abuse differently across sex, impacted both by developmental sex differences and hormonal status. Therefore, future research efforts will need to test this hypothesis in a systematic way.

## Conclusions

Melanin-concentrating hormone is an integral neurotransmitter system regulating physiology and motivated behavior and has important, varied effects based on sex. This neuropeptide stimulates feeding via nutrient detection and exerts this control in a sex-dependent manner. MCH’s effects on feeding appear to be under the inhibitory influence of the estrogen hormone system, influenced both by the amount of circulating estrogens and estrogen receptor density. As well, there appears to be a strong interaction between sexual development, the adult effects of sex hormones, and MCH.

Additionally, MCH activity also regulates multiple components of maternal behavior. Some of these behaviors are likely controlled by female-specific hypothalamic neurons that only express MCH during periods of maternal behavior. Specifically, MCH expression in these neurons correlates with the development of the pup, through the onset of maternal behaviors like nest building, and persists through the last maternal behavior to disappear, lactation. Moreover, constitutive MCH circuits appear to modulate olfactory-dependent parental behavior in both male and female rats, as well as food-seeking behavior. While future research will be needed to fully delineate the functional role of both transient and constitutive MCH neuronal populations on parentally motivated behaviors, it is possible that transient MCH expression seen only in female rats is part of a central energy homeostasis “infrastructure” that supports energy intensive maternal behavior. In contrast to maternal behavior, the intersection of sex, drugs of abuse, and MCH has only recently begun to be investigated systematically, even though sex as a factor has been known for some time to have an outsized influence on alcohol, methamphetamine, and nicotine seeking and taking behavior. While our recent work has highlighted that sex has a role in how MCH influences the behavioral symptoms of chronic nicotine exposure, we are at the initial stages of understanding what role central MCH circuits have on the symptoms of substance use disorder. This intersection may be important to fully delineate because MCH may be part of the neural substrate contributing to the changes in the motivation for drugs of abuse seen in females during pregnancy and maternal behavior. Therefore, sex differences discovered in MCH’s regulation of maternal behavior may inform our research on this system’s regulation of substance use disorder.

Taken together, MCH activity is highly involved in various forms of motivated behavior, with many clear examples of sex as a contributing factor. While feeding, maternal behavior, and olfaction have investigated sex differences directly, there is a notable paucity of data with the individual components that make up drug seeking and drug taking. Importantly, how MCH regulates substance use disorder-related behavior considering sex variables has only recently been explored, and many important questions remain to be resolved. This is an important part of understanding the biological influence of sex on MCH’s role in underpinning motivated behavior and the behavioral symptoms of substance use disorder in humans. In advancing toward more personalized medicine, it is crucial to gain a better understanding of sex as a biological variable to improve clinical treatment outcomes for substance use disorder in people.

## Data Availability

Data sharing not applicable to this article as no datasets were generated or analyzed during the current study.
